# Effectiveness of Orthopantomograms as a Screening Tool for Osteoporosis: A Case-Control Study

**DOI:** 10.7759/cureus.45702

**Published:** 2023-09-21

**Authors:** Dhanya M, Jayanth Kumar, Karthikeyan Ramalingam

**Affiliations:** 1 Oral Medicine and Radiology, Saveetha Dental College and Hospital, Chennai, IND; 2 Oral Pathology and Microbiology, Saveetha Dental College and Hospital, Chennai, IND

**Keywords:** antegonial angle, osteoporosis, drug-induced osteoporosis, postmenopausal women, orthopantomogram, mandibular cortical width

## Abstract

Introduction

Osteoporosis is a disease that is characterised by low bone mineral density (BMD), and loss of structural and biomechanical properties that are essential in maintaining bone homeostasis. Osteoporosis is diagnosed by clinical measurement of BMD and is the best predictor of osteoporosis. The study was conducted with the aim of assessing the effectiveness of orthopantomogram (OPG) as a screening tool for osteoporosis in postmenopausal women and chronic drug users.

Objectives

The primary objective of the current study was to assess the mandibular cortical width and antegonial index in postmenopausal women and chronic drug users, the secondary objective was to compare the mandibular cortical width and antegonial index of postmenopausal women and chronic drug users with that of the control group (healthy individual).

Methods

Three groups were taken in this study with a sample size of 300 with 100 OPG in each group. The groups categorised in the study were postmenopausal women, patients under drugs (glucocorticoids, proton pump inhibitor, anti-epileptic drugs, selective serotonin reuptake inhibitor) and the control group and the parameters assessed were antegonial index and mandibular cortical width.

Results

Results were tabulated and analysed using Statistical Package for the Social Sciences (IBM SPSS Statistics for Windows, IBM Corp., Version 26.0, Armonk, NY). The normality tests Kolmogorov-Smirnov and Shapiro-Wilks test results reveal that the variables (both indices) follow the normal distribution. The mandibular cortical width was 3.44, 2.66 and 2.96 in the normal, postmenopausal women and women on drugs respectively. The antegonial index was 163.5, 157.2 and 158.8 in the normal, postmenopausal women and women on drugs respectively.

Conclusion

From the above results, it is evident that there is a statistically significant reduction in antegonial index and mandibular cortical width in postmenopausal women compared to normal individuals. Alterations of this value are suggestive that early pre-clinical changes of osteoporosis can be detected in the high-risk group using OPG.

## Introduction

Osteoporosis is a chronic degenerative disorder commonly seen in postmenopausal women. The disease causes a decrease in bone mass, changes in the microarchitectural design and fragility of the bone and leads to fractures. The diagnosis of osteoporosis is based on various radiological parameters. Osteoporosis is derived from the Greek word “osteon” meaning bone, “pores” meaning small passage or pore, and “osis” meaning condition [1]. One of the key features of osteoporosis is the thinning of the bone trabeculae due to osteoclastic activity leading to a decrease in bone strength and an increased tendency for fractures. In the cortical region there can be a tunneling resorption leading to thinning of the bone cortex [2].

According to the World Health Organization (WHO) formulated in the year 1994, they defined osteoporosis as a systemic disease characterized by low mineral density, deterioration of bone structure and with increased bone fragility [3]. The risk factors of osteoporosis include poor health, low BMI (body mass index), low intake of calcium supplements, and prolonged use of steroids exceeding a duration of three months. The technique sensitive - dual-energy X-ray absorptiometry (DEXA) - is the best available technique in assessing the density at coxa which detects the early changes or signs of osteoporosis [2,3]. WHO defines osteoporosis based on the T-score. The T-score is a comparison of bone mineral density (BMD) with age-matched adults. If the T-score is less than -2.5 (implying that the BMD is less than 2.5 times the mean BMD of age-matched adults), the individual is designated as having osteoporosis. A score range of -1.0 to -2.5 is pre-osteoporosis or osteopenia. A score greater than -1 is designated as normal [3]. Osteoporosis leads to many types of fractures, of which the spine, hip, and shoulder; which are more susceptible in women. Literature also reveals that hormonal changes play a vital role in osteoporosis [2,3]. Recent evidence suggests that personal histories such as tobacco smoking, chewing betel nuts and alcohol consumption have been observed as risk factors that could alter the serum levels of osteoprotegerin [3]. A reduction of the protein osteoprotegerin (which slows down bone resorption) can increase bone resorption [4].

Orthopantomography (OPG) which is used in routine dental screening. It has also been used in the past as a screening tool for osteoporosis. This was a cheaper alternative for DEXA [5]. There were several indices used for the assessment of osteoporosis like total teeth present in the dentition, extent of bone resorption, anatomical width of the lamina dura and inferior cortical width of the mandible [6]. From the above the inferior cortical width of the mandible also known as the mandibular cortical width index has been the most studied index and has been used for the assessment of osteoporosis [7]. There have been several studies conducted across ethnic groups to study the cortical width and compare it with standard assessment tests for osteoporosis [8,9]. In a study by Taguchi et al. [10], it was found that the radiomorphometric indices were reliable markers for osteoporosis. In addition to the radiomorphometric indices, there have been some parameters such as incisure depth of the mandible which has also been found to be reduced in patients with osteoporosis [11]. The radio morphometric features - the mandibular cortical width, antegonial index, thin and porous mandibular cortical width in the mandibular cortical index in a panoramic radiograph - can be valuable markers of osteoporosis [12,13].

Unlike the radio morphometric indices, many oral changes have also been associated with osteoporosis which includes loss of teeth, temporomandibular dysfunction, gingival bleeding, ill-fitting dentures and increased probing depth [14]. There are various clinical assessment tools for osteoporosis which include the Osteoporosis Index of Risk (OSIRIS), Osteoporosis Self-Assessment Tool (OST), Simple Calculated Osteoporosis Risk Estimation (SCORE), Osteoporosis Risk Assessment Instrument (ORAI) [14]. Prior literature surveys revealed that the usage of drugs like anti-epileptics, glucocorticoids and anti-psychotics have been known to cause bone mineral loss [3,15].

The study aimed to assess the effectiveness of an orthopantomogram (OPG) as a screening tool for osteoporosis in postmenopausal women and chronic drug users like glucocorticoids, proton pump inhibitors and anti-epileptic drugs. The rationale for choosing this group of drugs is that these drugs have a tendency to cause osteoporosis.

The principal objective of this current study was to assess the mandibular cortical index and antegonial index in postmenopausal and chronic drug users and succedaneous objectives were to compare the mandibular cortical index and antegonial index with that of controls (healthy controls).

## Materials and methods

This is a retrospective case-control study carried out from the radiographic archives of the OPG. The study was approved by the Institutional Ethics Committee of Saveetha Dental College with the approval number IHEC/SDC/OMED-2103/22/175. The study samples were randomly selected among patients reporting to the Department of Oral Medicine and Radiology from the year 2019-2022 in a private institution. The OPGs were collected from the archives in the Digital Imaging in COMunication (DICOM) Format. The images were exported to Jpeg format in 1:1 orientation and the radio morphic indices were analysed using ImageJ (Ver 1.54, National Institute of Health, University of Wisconsin, USA) by the primary researcher. The inclusion and exclusion criteria according to each group are given in Table 1.

**Table 1:** Inclusion and exclusion criteria

Inclusion Criteria	Exclusion Criteria
Group 1	Common to all groups
Premenopausal women	Patient-specific criteria
Group 2	Patients with surgical defects
Postmenopausal women as confirmed by their history	Patients who had undergone chemotherapy
Group 3	Patients who had undergone radiotherapy
Anti-eplieptic drug users (carbamazepine)	Radiograph-specific criteria
Long-term steroid users (> 3 months) (methylprednisolone)	Images with distortion
Anti-psychotic medications (tryptomer) Images with poor patient positioning	Images with poor patient positioning

With the above-mentioned inclusion and exclusion criteria and based on convenience sampling a total of 300 OPGs were selected for the study. The control group included women in the premenopausal stage. Based on the collected OPG, they were categorised into the following groups as given in Table 2.

**Table 2:** Groups and sample sizes

Groups	Sample Size	Sample Population
Group 1	100	Control group
Group 2	100	Postmenopausal women
Group 3	100	Patients under anti-epileptics, proton pump inhibitors, anti-psychotics

**Figure 1:**
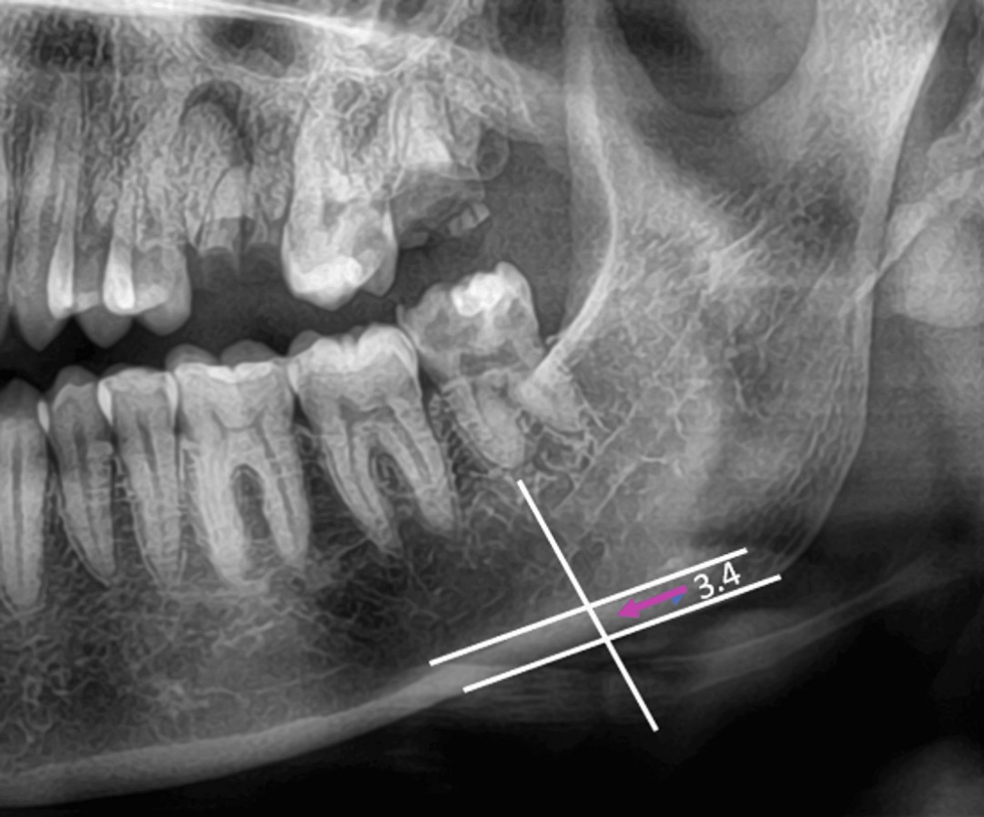
Mandibular cortical width - Group 1 - Controls

**Figure 2:**
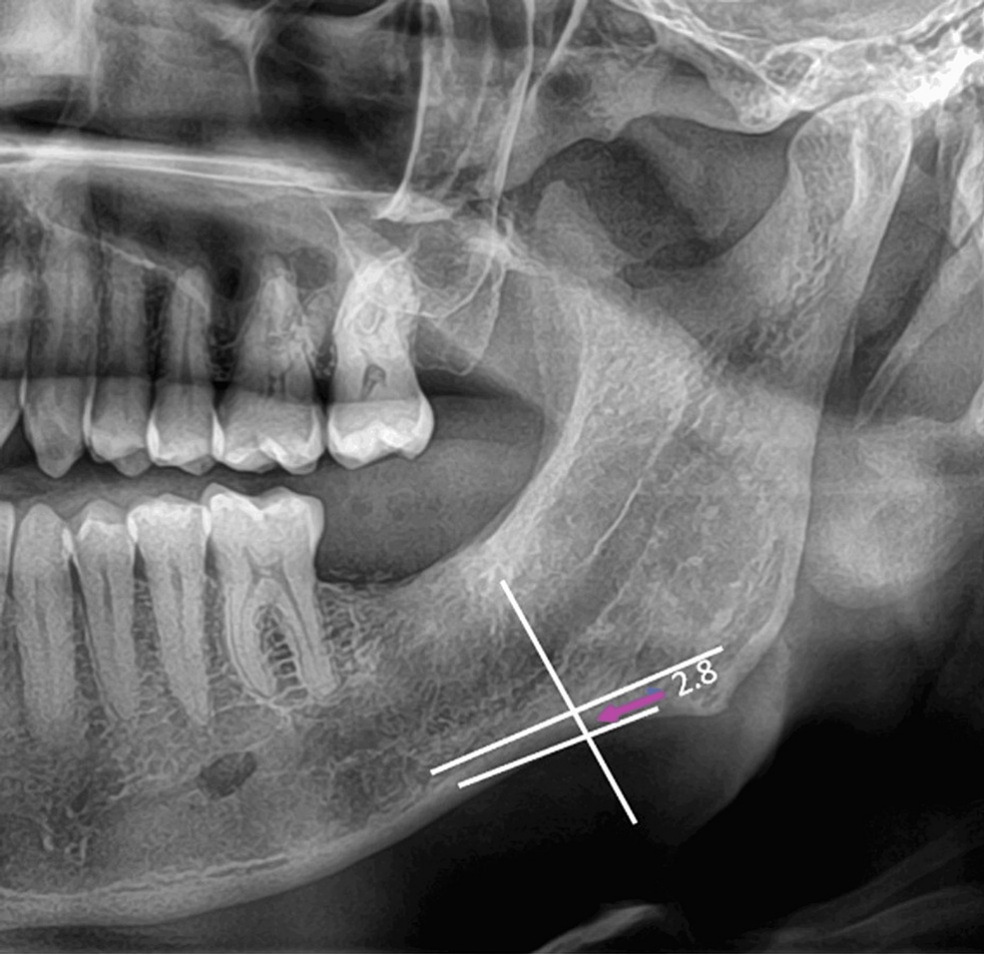
Mandibular cortical width - Group 2 - Postmenopausal women

**Figure 3:**
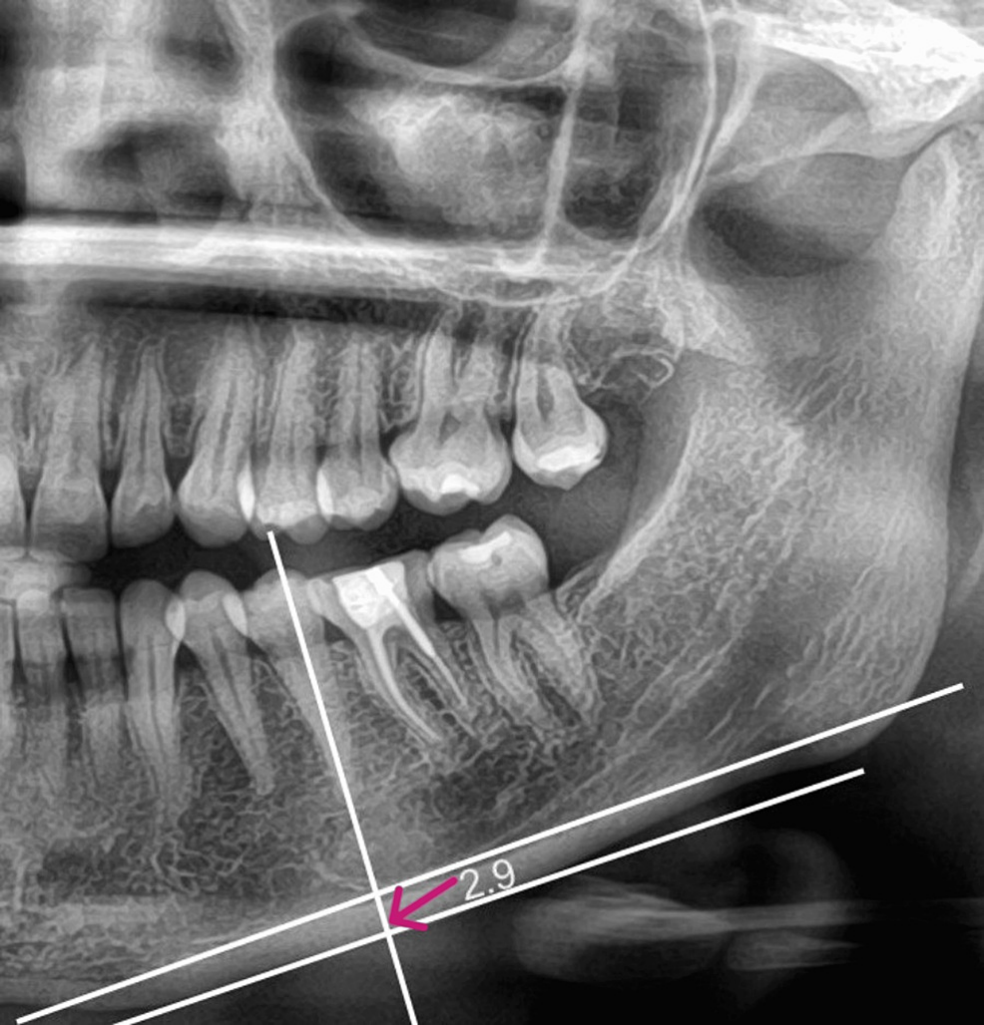
Mandibular cortical width - Group 3 - Women using long-term medication

For the study, the following parameters were measured. Parameters that were evaluated in a panoramic radiograph are panoramic mandibular cortical width and antegonial angle. The mandibular cortical width of the cortex is measured at the mental foramen as a line parallel to the long axis of the mandible and tangential to the inferior border of the mandible. All measurements were taken in mm (Figures 1, 2, 3).

**Figure 4:**
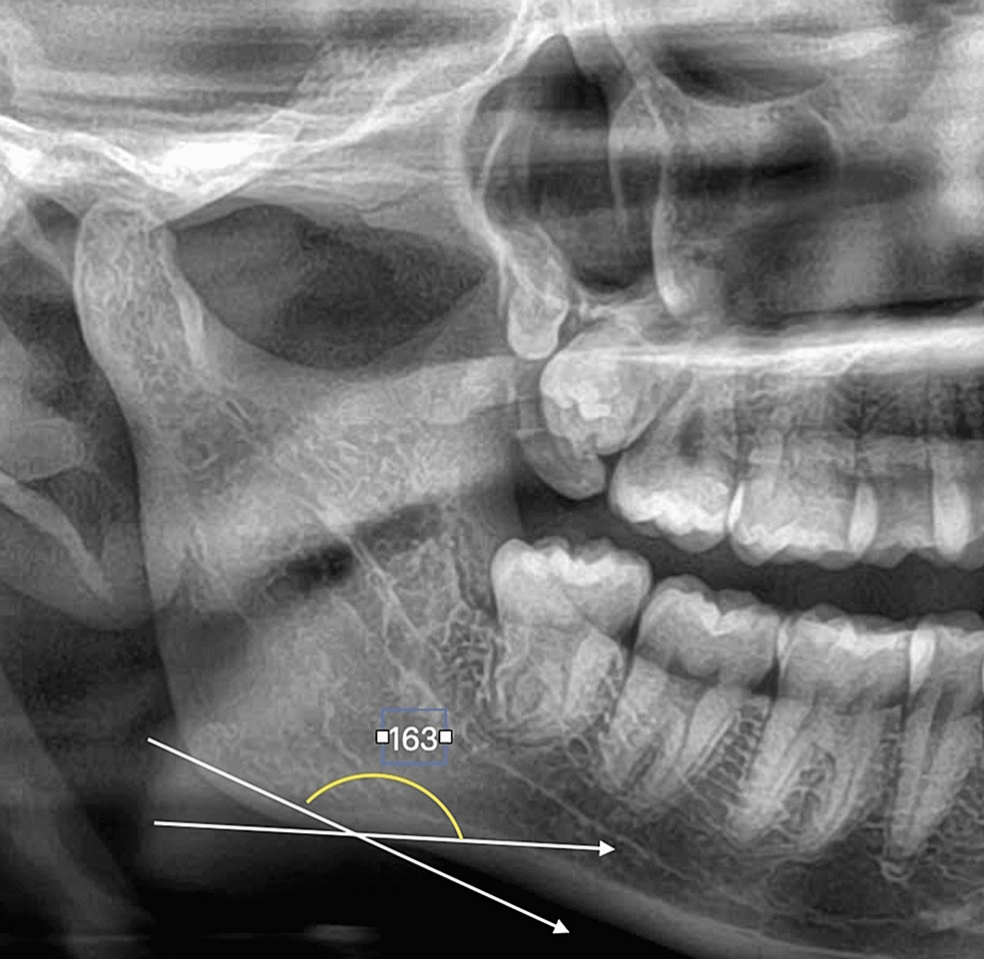
Antegonial angle - Group 1 - Controls

**Figure 5:**
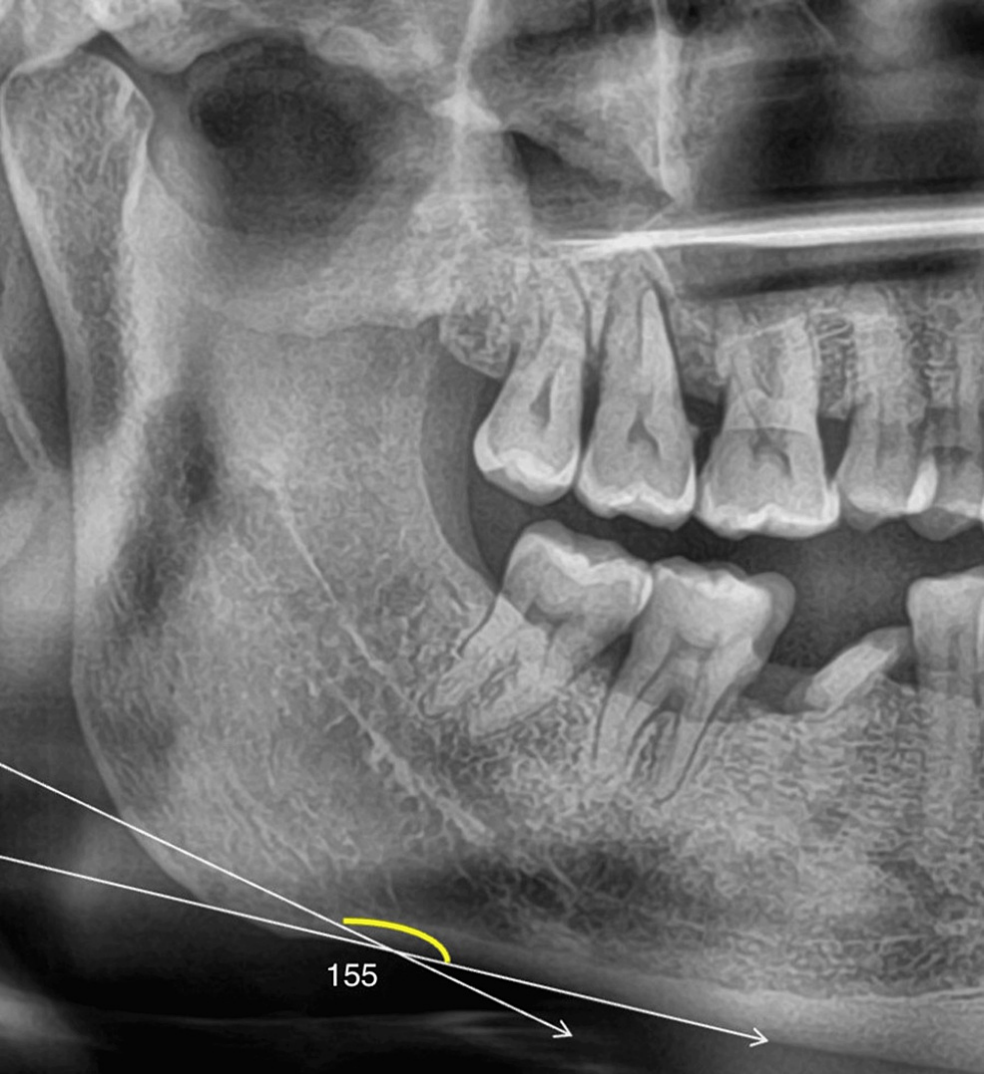
Antegonial angle - Group 2 - Postmenopausal women

**Figure 6:**
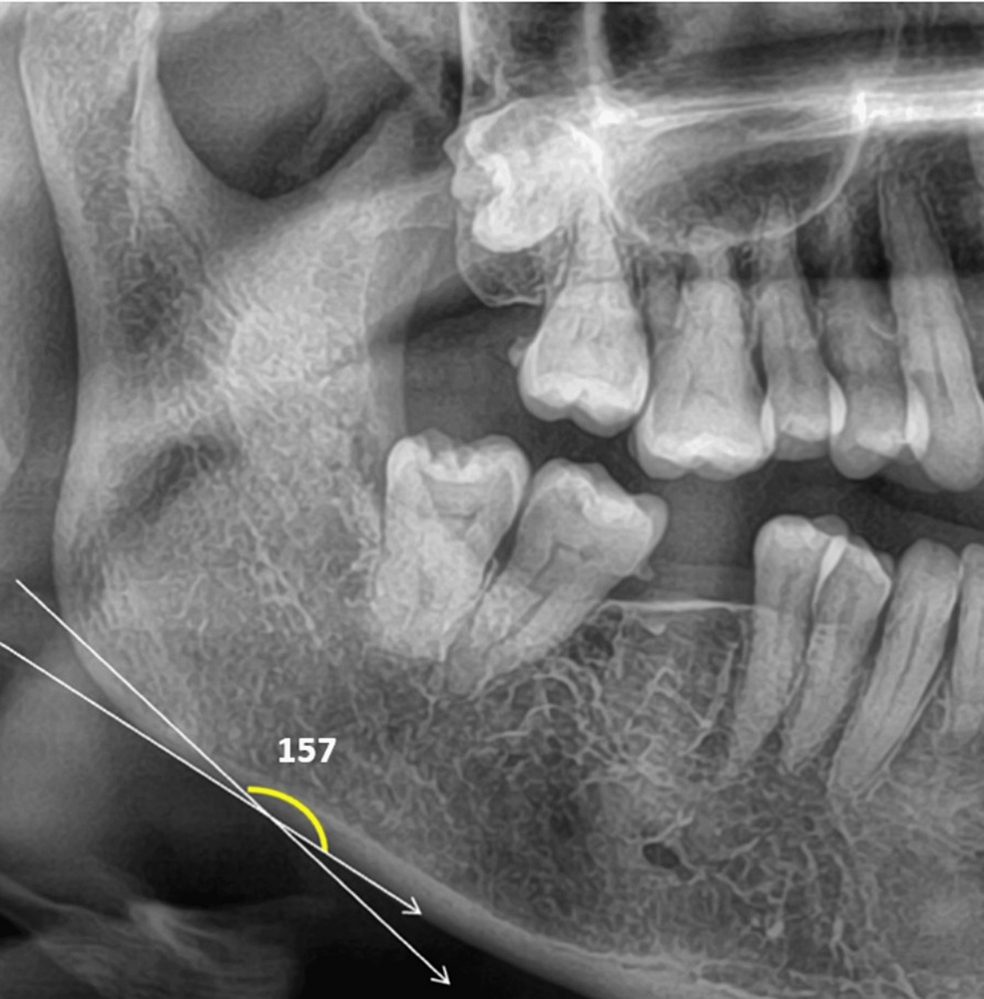
Antegonial angle - Group 3 - Women on long-term medication

Antegonial notch is measured by tracing two lines parallel to the lower cortical border at the antegonial region and measuring the angle of intersection at the deepest point of the antegonial notch, the normal angle is 163+/-2mm [3]. All measurements were taken in mm (Figures 4, 5, 6).

The collected data was analysed by Statistical Package for the Social Sciences (IBM SPSS Statistics for Windows, IBM Corp., Version 26.0, Armonk, NY).

## Results

The entire study population of 300 OPGs has been accounted for by statistical analysis. To analyse the data SPSS is used. The normality tests Kolmogorov-Smirnov and Shapiro-Wilks test results revealed that the variables (both indices) follow the normal distribution. Therefore, to analyse the data parametric statistical methods were applied. To compare the mean values between groups one-way analysis of variance (ANOVA) is applied followed by post hoc Tukey analysis. The level of significance was kept at 5%.

**Figure 7:**
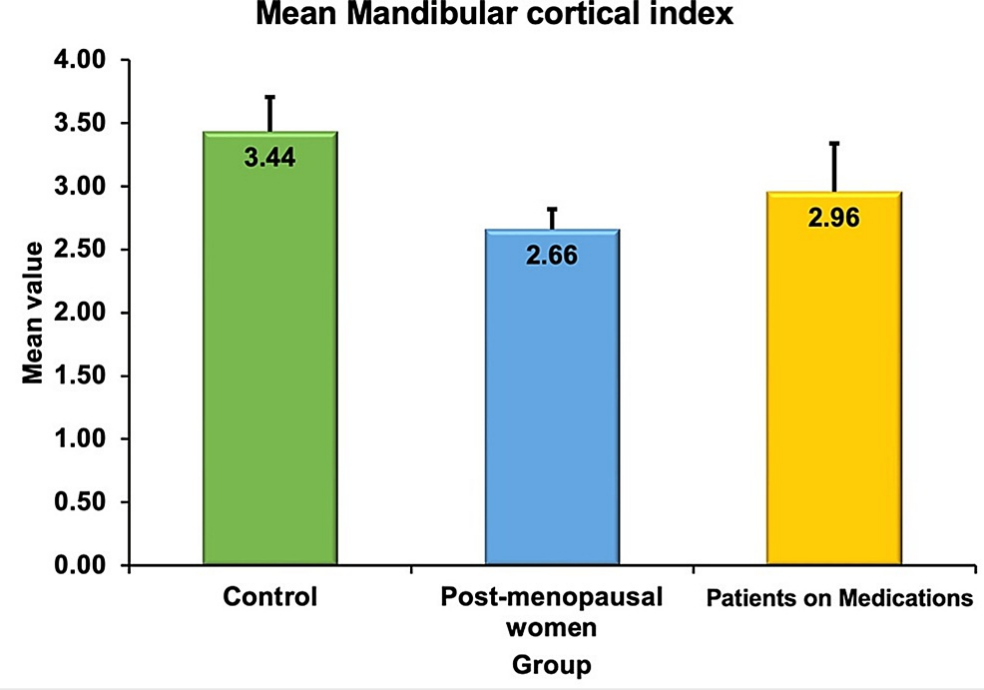
Bar graph with error bars showing the mean mandibular cortical width in three groups

The mandibular cortical width in mm was 3.43 ± 0.27 in group 1 (controls), 2.66 ± 0.16 in group 2 (postmenopausal women) and 2.96 ± 0.38 in group 3 (patients on medications like steroids, serotonin reuptake inhibitors and proton pump inhibitors). The above results are depicted in Figure 7. When one-way ANOVA was used to compare the mean between the groups the results were statistically significant (P = 0.042).

**Figure 8:**
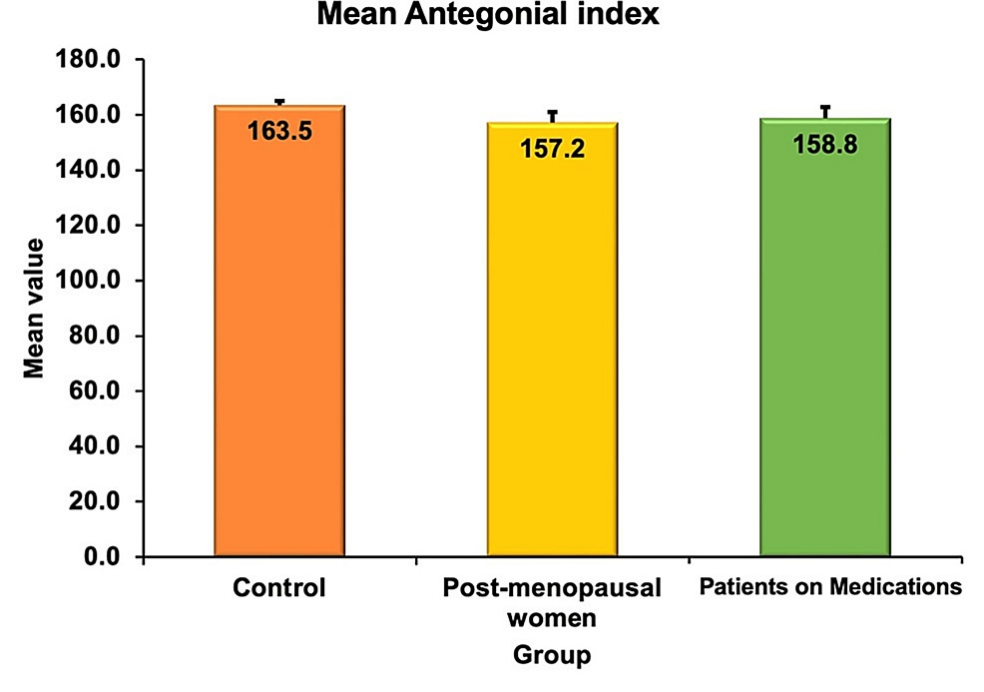
Antegonial index across three groups

The antegonial notch in mm was 163.51 ± 1.49, 157.21 ± 3.86 and 158.775 ± 4.06 in groups 1, 2 and 3 respectively (Figure 8). The results when compared by one-way ANOVA were statistically significant (P=0.025).

## Discussion

Osteoporosis is a degenerative bone disorder that causes thinning of the bone leading to pathological fractures. Though the confirmatory test is a DEXA scan, which is quite expensive, simple screening tools will help us in triaging for osteoporosis [16]. In a study by Hastar et al. [17], the mean mandibular cortical width in normals was 3.88 mm and that in osteoporosis individuals was 2.88 mm. In our study also the values were in line with the observation. From the current study, it is evident that postmenopausal women had significantly low bone density as evidenced by decreased mandibular cortical width and antegonial index when compared with controls and women under glucocorticoid therapy, antipsychotic therapy and anti-epileptic drugs.

In a study by Back et al. [15] they classified the cortex based on the cortical width into; mild, moderate and severe and they used 60 observers in assessing the OPG of the experimental individuals. The use of multiple observers leads to observer bias. In our study, we had a single observer who when in doubt had taken help from the other authors. In a study by Taguchi et al. [16] 450 postmenopausal women were taken as the experimental group and they took panoramic and cross-sectional images of cone beam computed tomography (CBCT) to measure the bone density, and later Taguchi et al. compared with the T-score of the spine. This study aimed to assess the effectiveness of the OPG but was not compared with the standard DEXA. However, this was a limitation of our study.

In a study by Ghapanchi et al. [18] the research explored the relationships between two panoramic radiography indices and BMD of postmenopausal women with osteopenia and osteoporosis. The study utilised receiver operating characteristic (ROC) curves with two parameters; the T-score of the cortical plate of the femur and that of the trabecular bone of the spine. In the current study, three radio morphometric indices were assessed using only panoramic images. In our study, we have proved that a decrease in both the radio morphometric indices was found in patients taking long-term steroid therapy, under medication for epilepsy and patients who are under anti-psychotic medication, especially selective serotonin reuptake inhibitors.

The study by Dumanic et al. [19] analysed the panoramic radiographs using software called DIGORA (DIG; Sordex Orion Corporation, Helsinki, Finland), as the current study the radio morphometric indices were analysed, in addition to the gonial index, and the mental index was assessed, the results showed a significant decrease in the mandibular cortical width with an increase in age. The software DIGORA is similar to our ImageJ software. There are studies in the literature stating that eliminating the risk factors such as tobacco and alcohol consumption, reducing caffeine ingestion, exercise, and supplementation with calcium and vitamin D reduced the fall in BMD [20].

Limitations

The key limitation of the study is that it was an unicentric study and changes in the radiomorphometric indices were not compared with the gold standard test of DEXA scan.

## Conclusions

The results are suggestive that OPGs show a change in the radiomorphometric indices between the three groups. These indices of mandibular cortical width and antegonial index are well-established markers for osteoporosis. In our study, we did notice that mandibular cortical width provided a substantial reduction in width compared to osteoporosis and the group in which women were on long-term medications. However, we need a well-exposed radiograph without geometric distortions to do the assessment for osteoporosis. A cost-effective OPG can be used as a screening tool for comparing between the high-risk groups.

## References

[1] JM Lane, Russel Russel, SM Khan (2000). Osteoporosis. Clini Orthop Relat Res.

[2] Myers ER, Wilson SE (1997). Biomechanics of osteoporosis and vertebral fracture. Spine (Phila Pa 1976).

[3] Camacho PM, Petak SM, Binkley N (2020). American Association of Clinical Endocrinologists/American College of Endocrinology clinical practice guidelines for the diagnosis and treatment of postmenopausal osteoporosis-2020 update. Endocr Pract.

[4] Abdi S, Binbaz RA, Mohammed AK (2021). Association of RANKL and OPG gene polymorphism in Arab women with and without osteoporosis. Genes (Basel).

[5] Vlasiadis KZ, Skouteris CA, Velegrakis GA, Fragouli I, Neratzoulakis JM, Damilakis J, Koumantakis EE (2007). Mandibular radiomorphometric measurements as indicators of possible osteoporosis in postmenopausal women. Maturitas.

[6] Pallagatti S, Parnami P, Sheikh S, Gupta D (2017). Efficacy of panoramic radiography in the detection of osteoporosis in post-menopausal women when compared to dual energy X-ray absorptiometry. Open Dent J.

[7] Dwivedi H, Singh B, Gupta P (2021). Correlation between Radiomorphometric indices and edentulous mandibular arches to diagnose osteoporosis using orthopantomogram in West Bengal state in India. J Contemp Dent Pract.

[8] Balto KA, Gomaa MM, Feteih RM, AlAmoudi NM, Elsamanoudy AZ, Hassanien MA, Ardawi MM (2018). Dental panoramic radiographic indices as a predictor of osteoporosis in postmenopausal Saudi women. J Bone Metab.

[9] Isales CM (2007). Role of the oral and maxillofacial surgeon in the diagnosis and treatment of patients with osteoporosis. Oral Maxillofac Surg Clin North Am.

[10] Taguchi A, Asano A, Ohtsuka M (2008). Observer performance in diagnosing osteoporosis by dental panoramic radiographs: results from the osteoporosis screening project in dentistry (OSPD). Bone.

[11] Rehman DE, Qureshi S, Abdul Haq A (2014). Early detection of osteoporosis from incisure depth of human mandible in an orthopantomogram. J Pak Med Assoc.

[12] Kumar DP, Jayachandran S, Thilagavathy N (2021). Diagnostic validity of orthopantomogram compared to dual energy X-ray absorptiometry scan in detecting osteoporosis. Ann Natl Acad Med Sci.

[13] Taguchi A (2010). Triage screening for osteoporosis in dental clinics using panoramic radiographs. Oral Dis.

[14] Navabi N, Motaghi R, Rezazadeh M, Balooch H (2018). Relationship between two panoramic radiography indices and bone mineral density of postmenopausal women with osteopenia and osteoporosis. J Dent (Shiraz).

[15] Bäck K, Ahlqwist M, Hakeberg M, Björkelund C, Dahlström L (2017). Relation between osteoporosis and radiographic and clinical signs of osteoarthritis/arthrosis in the temporomandibular joint: a population-based, cross-sectional study in an older Swedish population. Gerodontology.

[16] Taguchi A, Tanaka R, Kakimoto N (2021). Clinical guidelines for the application of panoramic radiographs in screening for osteoporosis. Oral Radiol.

[17] Hastar E, Yilmaz HH, Orhan H (2011). Evaluation of mental index, mandibular cortical index and panoramic mandibular index on dental panoramic radiographs in the elderly. Eur J Dent.

[18] Ghapanchi J, Zahed M, Haghnegahdar A, Niakan N, Sadeghzadeh A (2018). Osteoporosis and jaw abnormalities in panoramic radiography of chronic liver failure patients. Biomed Res Int.

[19] Savic Pavicin I, Dumancic J, Jukic T, Badel T, Badanjak A (2014). Digital orthopantomograms in osteoporosis detection: mandibular density and mandibular radiographic indices as skeletal BMD predictors. Dentomaxillofac Radiol.

[20] Papamanthos M, Varitimidis S, Dailiana Z, Kogia E, Malizos K (2014). Computer-assisted evaluation of mandibular cortical width (MCW) index as an indicator of osteoporosis. Hippokratia.

